# Antibacterial Properties and Mechanisms of Action of Sonoenzymatically Synthesized Lignin-Based Nanoparticles

**DOI:** 10.1021/acsami.2c05443

**Published:** 2022-08-12

**Authors:** Angela Gala Morena, Arnau Bassegoda, Michal Natan, Gila Jacobi, Ehud Banin, Tzanko Tzanov

**Affiliations:** † Group of Molecular and Industrial Biotechnology, Department of Chemical Engineering, Universitat Politècnica de Catalunya, Rambla Sant Nebridi 22, Terrassa 08222, Spain; ‡ The Mina and Everard Goodman Faculty of Life Sciences, Bar-Ilan University, Bldg 206, Ramat-Gan 82900, Israel

**Keywords:** lignin, antibacterial, nanoparticle, enzymatic grafting, laccase, sonochemistry, antimicrobial resistance

## Abstract

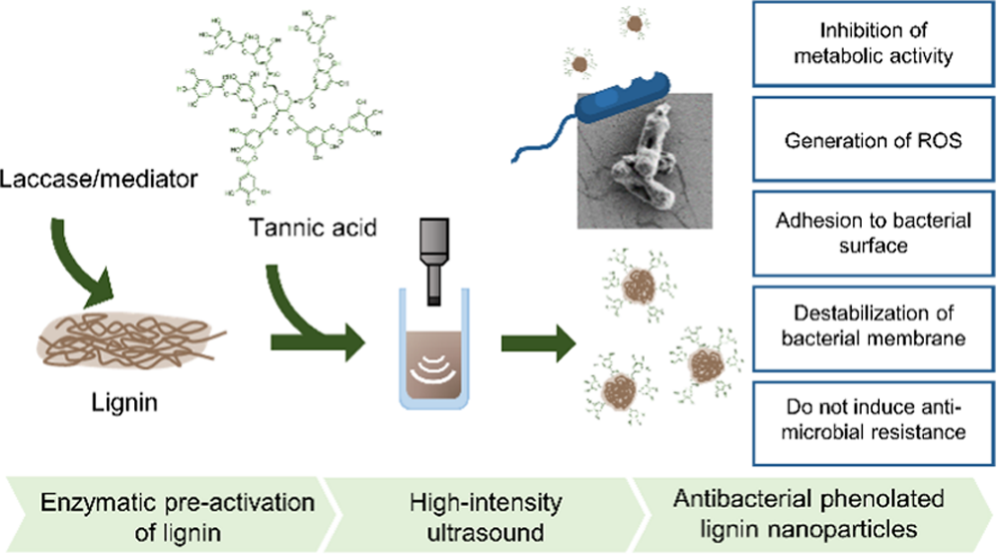

In recent years, lignin has drawn increasing attention for different applications due to its intrinsic antibacterial and antioxidant properties, coupled with biodegradability and biocompatibility. However, chemical modification or combination with metals is usually required to increase its antimicrobial functionality and produce biobased added-value materials for applications wherein bacterial growth should be avoided, such as biomedical and food industries. In this work, a sonoenzymatic approach for the simultaneous functionalization and nanotransformation of lignin to prepare metal-free antibacterial phenolated lignin nanoparticles (PheLigNPs) is developed. The grafting of tannic acid, a natural phenolic compound, onto lignin was achieved by an environmentally friendly approach using laccase oxidation upon the application of high-intensity ultrasound to rearrange lignin into NPs. PheLigNPs presented higher antibacterial activity than nonfunctionalized LigNPs and phenolated lignin in the bulk form, indicating the contribution of both the phenolic content and the nanosize to the antibacterial activity. Studies on the antibacterial mode of action showed that bacteria in contact with the functionalized NPs presented decreased metabolic activity and high levels of reactive oxygen species (ROS). Moreover, PheLigNPs demonstrated affinity to the bacterial surface and the ability to cause membrane destabilization. Antimicrobial resistance studies showed that the NPs did not induce resistance in pathogenic bacteria, unlike traditional antibiotics.

## Introduction

Lignin is one of the main macromolecules in the lignocellulosic biomass and the most abundant sustainable source of aromatic compounds. Annually, the paper industry generates around 70 million tons of this biopolymer as a byproduct that is mainly used as fuel or discarded as a waste liquid.^1^ Despite the huge potential of lignin for replacing synthetic chemicals, only 2–5% of this biopolymer in its macromolecular form is commercialized as an additive to formulate adhesives and polyurethanes or as a surfactant for colloidal suspensions.^2^

In the previous years, lignin has gained interest in industrial and biomedical applications, for instance, in the synthesis of chemicals, polymers, and bioactive nanoparticles, owing to the large variety of functional groups it contains, including phenolic and aliphatic hydroxyls, carboxylic, carbonyl, and methoxyl groups, which confer antibacterial, antioxidant, and UV stabilizing properties.^2,3^ In particular, nanoformulation of lignin yields nanomaterials with higher reactivity in comparison with their bulk counterparts, and their addition to composites is known to impart improved mechanical and bioactive properties.^4^ In this line, lignin nanoparticles (LigNPs) have been used in polymeric materials as mechanical reinforcement,^5^ as UV absorbents,^6^ as antibacterial and antioxidant agents in food packaging,^7^ and as carriers for drug delivery.^8^

Different green methods such as solvent exchange,^9^ water-in-oil microemulsion,^10^ and ultrasonication^11,12^ are used to produce LigNPs for biomedical and food applications wherein biocompatibility and biodegradability are crucial features. However, chemical modification of lignin or its combination with metals is usually a requirement to potentiate the functionalities of the NPs and extend their application range.^13,14^ Several techniques have been used to enhance the antibacterial and antioxidant properties of lignin and LigNPs, including phenolation^15,16^ and amination.^17^ Traditional functionalization strategies often involve toxic reagents or metal catalysts and require harsh conditions. An eco-friendly alternative is the use of enzymatic catalysis for grafting functional molecules onto lignin to achieve improved performance in specific applications. This biotechnological approach provides clear environmental advantages over chemical modification, allowing to work under mild conditions and avoiding the use of hazardous chemicals, ultimately reducing the amount of toxic residues.^18^

Laccases are oxidoreductases found in plants, fungi, and bacteria that catalyze the oxidation of a wide range of substrates, including phenolic lignin-related compounds. Once oxidized, the phenolic group yields radicals that rapidly form reactive phenoxy radicals, which are able to form covalent bonds with other molecules. The industrial relevance of these enzymes is associated with multiple applications, e.g., pulp bleaching, bioremediation, and biosensing.

In view of the growing concern about the rapid surge of antimicrobial resistance (AMR) in bacteria, there is an urgent need to develop environmentally friendly antimicrobial agents as an alternative to classic antibiotics adopting green chemical approaches.^19^ The phenolic groups present in lignin endow this biopolymer with intrinsic antibacterial capacity.^20^ In this work, we aim to enhance the antibacterial activity of lignin by increasing its phenolic content and formulating nanoparticles that could find applications as antibacterial agents in food packaging and biomedical fields. The rationale of our study is based on the enzymatic preactivation of lignin using acetosyringone as an effective laccase mediator^21^ to expand the oxidative action of laccase to molecules that are sterically inaccessible to the enzyme.^22^ Then, the addition of the phenolic compound tannic acid (TA) in the presence of laccase and the application of ultrasound would simultaneously (i) initiate cross-linking reactions between TA and lignin and (ii) form NPs. Nonfunctionalized lignin nanoparticles (LigNPs) and phenolated bulk lignin (PheLig) were also prepared to evaluate the contribution of the nanoform and the phenolic content to the antibacterial activity. The potential cytotoxic effects of the NPs were evaluated *in vitro* on human fibroblast and keratinocyte cells. Finally, to elucidate the antibacterial mechanism of action of the PheLigNPs, the interaction between the NPs and bacterial surfaces, and the influence of NPs on the generation of reactive oxygen species (ROS) and metabolic activity of bacteria were studied.

## Experimental Section

### Materials, Enzymes, and Bacteria

Protobind 6000 lignin was purchased from Green Value (Switzerland). Tannic acid (TA), gallic acid (GA), and 3′,5′-dimethoxy-4′-hydroxyacetophenone (acetosyringone) were obtained from ACROS Organics (Belgium). Folin–Ciocalteu phenol reagent, resazurin sodium salt, phosphate-buffered saline (PBS), nutrient broth (NB), Luria–Bertani (LB) with agar, Baird-Parker agar, Coliform ChromoSelect agar, cetrimide agar, and Dulbecco’s modified Eagle’s medium (DMEM) were purchased from Sigma-Aldrich (Spain). AlamarBlue cell viability reagent, molecular probe 2′,7′-dichlorodihydrofluorescein diacetate (H_2_DCFDA), and Live/Dead BacLight kit (Molecular probes L7012) were obtained from Invitrogen, Life Technologies Corporation (Spain). Novozymes (Denmark) supplied fungal laccase Novozym 51003 from *Myceliophthora thermophila* (EC 1.10.3.2) with an activity of 1322 U·mL^–1^ defined as the amount of enzyme converting 1 μmol ABTS to its cation radical (ε_436_ = 29,300 M^–1^·cm^–1^) in 0.05 M sodium acetate buffer pH 5 at 25 °C. Phosphatidylethanolamine (PE, #840027) and phosphatidylglycerol (PG, #841188) extracted from *Escherichia coli* were provided by Avanti Polar Lipids. Bacterial strains (*Staphylococcus aureus* ATCC 25923, *Bacillus cereus* 14579, *E. coli* ATCC 25922, *Pseudomonas aeruginosa* ATCC 10145), and human cell lines (fibroblast ATCC-CRL-4001, BJ-5ta, and keratinocyte HaCaT) were purchased from the American Type Culture Collection (ATCC LGC Standards, Spain). Ciprofloxacin and ampicillin were purchased from Fluka and Duchefa, respectively. Ultrapure Millipore water (18.2 MΩ·cm resistivity) was used in all experiments.

### Synthesis of PheLigNPs

Lignin (10 mg·mL^–1^) was soaked in sodium acetate buffer (50 mM, pH 5), where acetosyringone was previously dissolved (1.5 mg·mL^–1^). Laccase at a final concentration of 13.2 U·mL^–1^ was added, and the mixture was stirred for 1 h at 50 °C to preactivate lignin. Then, 10 mg·mL^–1^ tannic acid was added, and the solution was subjected to ultrasound (20 kHz, 50% amplitude, Ti-horn) for 1 h at 50 °C to produce the PheLigNPs (VCX 750 ultrasonic processor, Sonics). The mixture was centrifuged at 18,000*g* for 20 min to remove unreacted tannic acid molecules present in the supernatant. The pellet was resuspended in water and concentrated ten times; then, disaggregation of the particles was achieved by applying low-intensity ultrasound. At last, the NPs were centrifuged at 500*g* for 10 min to remove larger aggregates. The PheLigNPs were stored at 4 °C.

The bulk phenolated lignin (PheLig) was prepared as previously described with some modifications.^23^ Briefly, preactivation of lignin with laccase was carried out as described above, followed by 2 h stirring with tannic acid at 50 °C. The LigNPs were synthesized by sonicating a solution of lignin in water (10 mg·mL^–1^) at 50% amplitude and 50 °C for 2 h, following an adapted protocol previously reported.^11^

### Characterization of PheLigNPs

The hydrodynamic size, polydispersity index (PDI), and ζ-potential of the NPs were measured using a Zetasizer Nano Z (Malvern Instruments Inc., U.K.). The phenolic content of lignin, phenolated lignin, LigNPs, and PheLigNPs was determined using the Folin–Ciocalteu phenol reagent as previously described.^23^ All samples were measured in triplicate, and the results were expressed in GA equivalents per gram of sample. Transmission electron microscopy (TEM) was used to study the morphology and distribution of the NPs by placing 10 μL of diluted sample onto holey carbon films on copper grids. The samples were observed using a JEOL JEM-2100 LaB6 microscope operating at an accelerating voltage of 200 kV. Nanoparticle size was measured using ImageJ software (version 1.52a). Fourier transform infrared (FTIR) spectra of lignin samples over the 600–4000 cm^–1^ range were collected by a PerkinElmer Spectrum 100 FTIR spectrometer (PerkinElmer, MA) with an attenuated total reflection (ATR) accessory of germanium crystal with a high-resolution index (4.0), performing 64 scans for each spectrum at 4 cm^–1^ resolution. The peak at 2920 cm^–1^, corresponding to the C–H stretching in aromatic methoxyl groups, was used for normalization.^24^ For the semiquantitative analysis, the relative intensities of the respective bands were calculated taking the band at 2920 cm^–1^ as the reference. The ratio of *A*_x_/*A*_2920_ was taken as the quotient between the specific intensity and the reference band.

### Determination of the Minimum Inhibitory Concentration (MIC)

The antibacterial activity of PheLigNPs, LigNPs, phenolated lignin, and lignin was assessed toward *S. aureus*, *B. cereus*, *P. aeruginosa*, and *E. coli* following the serial dilution method, and the minimum inhibitory concentration (MIC) was determined as previously described.^13^ Briefly, bacterial suspensions in NB at 10^5^–10^6^ colony forming units (CFU) per milliliter were incubated with lignin samples at different concentrations (5–0.3 mg·mL^–1^ diluted in NB) for 24 h at 37 °C with shaking. The optical density (OD) at 600 nm was measured to obtain the MIC.

### Kinetic Growth Curves of Bacteria Incubated with PheLigNPs

For the kinetic growth curves, bacteria grown overnight in NB were diluted in NB medium to an OD_600_ = 0.01 (∼10^5^–10^6^ CFU·mL^–1^). Then, 250 μL of bacterial suspension was incubated with 250 μL of lignin samples in 2 mL tubes at 37 °C with 230 rpm shaking. Growth controls were bacteria cultured in NB without lignin samples. After 0, 4, 8, and 24 h of incubation, samples were taken, and the number of survived bacteria (CFU·mL^–1^) was obtained using the drop plate method. Briefly, serial dilutions of the samples were performed in PBS, and 10 μL drops were plated in Baird-Parker, LB, cetrimide, and Coliform ChromoSelect agar plates for *S. aureus*, *B. cereus*, *P. aeruginosa*, and *E. coli*, respectively. After 24 h incubation of the agar plates at 37 °C, the grown colonies were counted.

### Cytotoxicity Assay

Cytotoxicity of the lignin samples was tested toward human cell lines (fibroblasts and keratinocytes) as described elsewhere.^13^ In brief, cells seeded in 96-well tissue culture-treated polystyrene plate (60,000 cells per well) were incubated for 24 h in the presence of lignin samples at different concentrations. The cell viability was determined using the AlamarBlue reagent and compared to that of cells incubated in absence of lignin samples (growth control, 100% cell viability).

### Determination of Metabolic Activity of Bacteria by the Resazurin Assay

The effect of PheLigNPs on the metabolic activity of bacteria was assessed using the resazurin method. Overnight bacterial inocula in NB were diluted to OD_600_ = 0.01 (∼10^5^–10^6^ CFU·mL^–1^) in NB. Then, 50 μL of bacteria was mixed with 50 μL of PheLigNPs (final concentration of 0.6 mg·mL^–1^). After 4 h of incubation at 37 °C and 230 rpm shaking, 10 μL of resazurin 100 μg·mL^–1^ was added and further incubated for 10 min. Finally, the fluorescence was measured at λ_ex/em_ = 520/590 nm. Bacteria grown in NB were used as a reference to calculate the metabolic activity
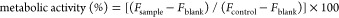
where *F*_sample_ is the fluorescence of bacteria incubated with PheLigNPs, *F*_blank_ is the fluorescence of NB media, and *F*_control_ refers to the fluorescence of bacteria grown in NB.

### Quantification of Reactive Oxygen Species Generation by Bacteria

The generation of reactive oxygen species (ROS) by bacteria in contact with PheLigNPs was studied using oxidation-sensitive probe H_2_DCFDA. For the assay, bacterial cultures of *S. aureus*, *B. cereus*, *P. aeruginosa*, and *E. coli* in NB were grown to an OD_600_ of ∼0.8 and exposed to PheLigNPs (2.5 mg·mL^–1^). After 30 min at 37 °C, the mixtures were centrifuged at 4000*g* and washed twice with PBS. The bacteria in the pellet were incubated with a solution of 20 μM H_2_DCFDA in PBS for 30 min in the dark. Then, the fluorescence was measured at λ_ex/em_ = 490/520 nm. Controls were bacterial dispersions incubated without PheLigNPs.

### Interaction of PheLigNPs with Bacteria Assessed by Scanning Electron Microscopy (SEM)

To study the interaction of PheLigNPs with bacteria and their effect on cell structure, bacterial cell cultures grown overnight in NB at 37 °C were diluted to an OD_600_ = 0.01 (∼10^5^–10^6^ CFU·mL^–1^) and mixed with PheLigNPs (final concentration 1.25 mg·mL^–1^). After 4 h incubation at 37 °C and 230 rpm, the volume was transferred to a 48-well plate containing silicon wafers. The samples were further incubated overnight at room temperature. Then, the liquid was carefully removed and the bacteria remaining in the grid were fixed overnight in a 2% paraformaldehyde- and 2.5% glutaraldehyde-buffered solution. The samples were dehydrated incubating the wafers with increasing concentrations of ethanol for 1 h each (25, 50, 75, and 100%). The samples were observed using a field-emission scanning electron microscope (SEM) at 1 kV (Merlin Zeiss).

### Interaction of PheLigNPs with Bacteria Assessed by a Quartz Crystal Microbalance

The interaction of PheLigNPs with bacteria was assessed using a quartz crystal microbalance with dissipation monitoring (QCM-D, E4 system, Q-Sense, Sweden), following a previously reported procedure with some modifications.^25^ The experiments were performed on gold sensors (QSX 301, Q-Sense, Sweden) at 37 °C. A peristaltic pump was used to flow the liquids at a constant rate of 20 μL·min^–1^. First, sterile PBS (100 mM, pH 7.4) was used for 30 min to establish a stable baseline. The deposition of bacteria on the sensor was achieved by circulating the *S. aureus* inoculum (OD_600_ = 0.2) in NB for 3 h. To remove the nondeposited bacteria, PBS was flowed through the system for 1 h and a second baseline was established. Thereafter, PheLigNPs (2.5 mg·mL^–1^ in PBS) were circulated for 45 min. Finally, the third baseline was established by further flowing PBS.

### Interaction of PheLigNPs with Bacterial Model Membranes Assessed by Langmuir Isotherms

The interaction of the NPs with bacterial model membranes was studied using Langmuir isotherms. A mixture of PE and PG in an 8:2 ratio (v/v) at 0.5 mg·mL^–1^ in chloroform was used to simulate the *E. coli* bacterial membrane. Monolayers were formed in a Langmuir trough equipped with two moving barriers (KSV NIMA Langmuir–Blodgett Deposition Troughs, model KN2002, Finland) at a constant temperature of 23 ± 1 °C. The surface pressure (π) was measured using a Wilhelmy plate connected to the trough. After cleaning the system with chloroform and water, the subphase (PBS or PBS with PLN) was added and 30 μL of lipids was gently added. After the chloroform from the lipid mixture was evaporated, the surface pressure–area per molecule (π–A) isotherm was recorded under a barrier closure rate of 15 cm^2^·min^–1^. The experiments were carried out three times, and one representative measure was reported.

### Determination of Membrane Integrity by Fluorescence Imaging

Membrane integrity of bacteria incubated with PheLigNPs was assessed using the Live/Dead BacLight bacterial viability kit according to the manufacturer’s instructions. Bacterial cell cultures grown overnight in NB at 37 °C were diluted to an OD_600_ = 0.01 (∼10^5^–10^6^ CFU·mL^–1^) and mixed with PheLigNPs (final concentration 0.6 mg·mL^–1^). After 24 h incubation at 37 °C and 230 rpm, the cells were washed by centrifugation at 10,000*g* for 2 min and the pellet was resuspended in 0.9% NaCl. The bacterial suspension was stained with an equal mixture of SYTO-9 and propidium iodide and observed using a fluorescence microscope (Nikon/Eclipse Ti-S, the Netherlands) after 15 min incubation in the dark. Bacteria grown in the absence of PheLigNPs were used as controls.

Damage to the bacterial membrane was also assessed by measuring the fluorescence of the suspensions using a microplate reader (Infinite M200, Tecan, Austria). The excitation wavelength was 485 nm for both dyes, and the fluorescence was read at 530 nm for SYTO-9 and at 645 nm for propidium iodide. Fluorescence values of untreated and treated bacteria without the dyes were used as blanks. The ratio of SYTO-9 to propidium iodide (R = emission SYTO-9/emission propidium iodide) was calculated.

### Resistance Development Assay

The ability of the PheLigNPs to cause resistance development among pathogenic bacteria was evaluated by determining first the MIC value using *S. aureus* ATCC 29213 and *E. coli* 25922. The stock solution of the NPs was diluted in 2-fold serial dilutions in LB medium in a 96-well plate (Greiner Bio-one), and bacteria were added to a final concentration of bacteria of 10^5^ CFU·mL^–1^. The control was bacteria treated with water. The bacterial growth was monitored by measuring the absorbance at OD_595_ using a microplate reader (Synergy 2, BioTek instruments). On the following day, each bacteria was serially passaged in 2-fold antibiotic or NPs gradients in a 96-well plate, performing a MIC assay as described above except that the bacteria concentration was set on 10^5^ CFU·mL^–1^ from the second growth cycle. At the end of each growth cycle (20–24 h) following the determination of the MIC, the culture in the highest drug concentration having turbidity, suggestive of bacterial growth, was taken and diluted at 1:50. The newly diluted bacterial suspension was grown overnight in a new 96-well plate, conducting a new MIC assay, following which the absorbance was monitored. This assay was conducted daily for a period of 30 days to determine the change in the MIC value of the antibiotic or the NPs.

## Results and Discussion

### Characterization of PheLigNPs

**Figure 1:**
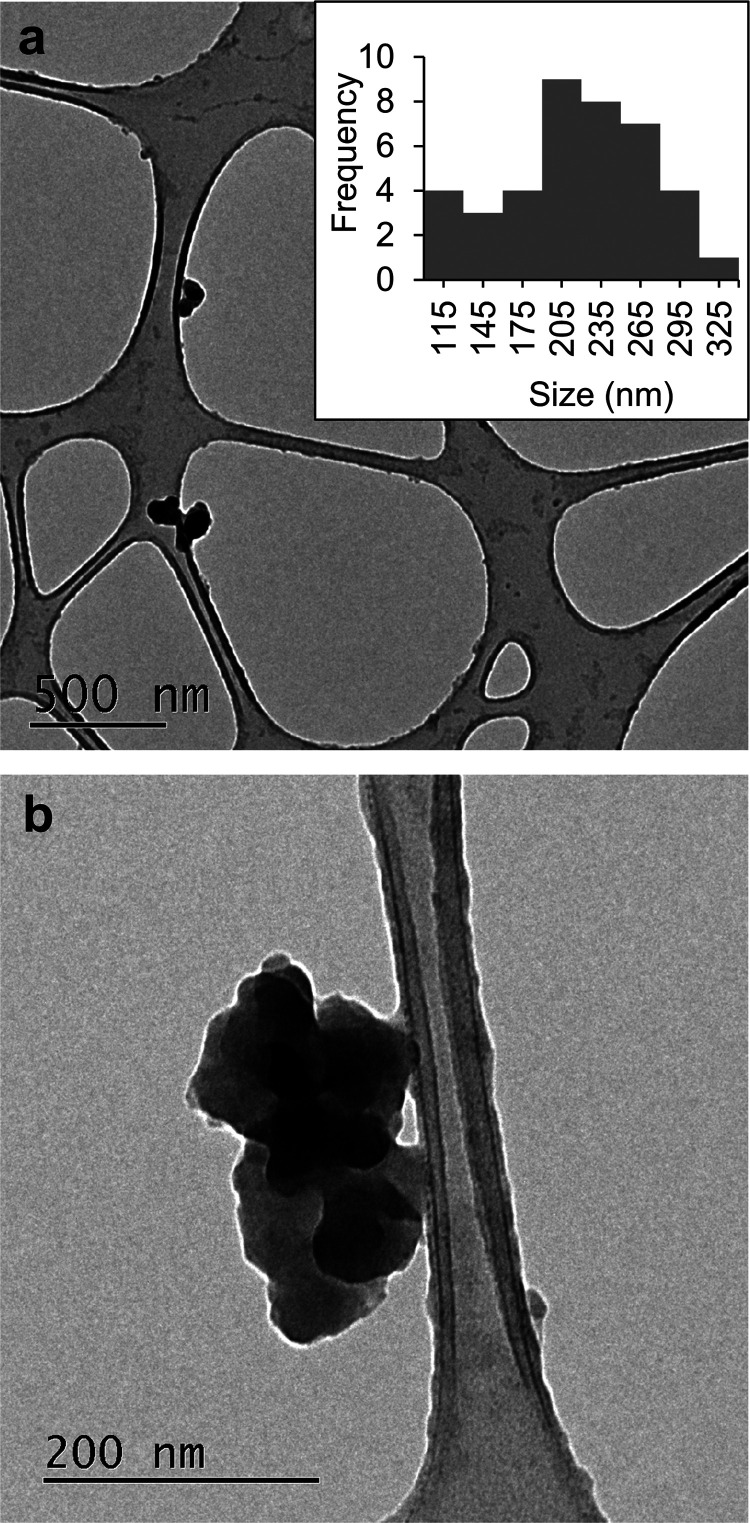
(a) TEM image of PheLigNPs at 4000× and size distribution of the particles (inset) and (b) TEM image of PheLigNPs at 20,000× magnification.

The combination of the laccase/mediator system to graft the natural phenolic compound tannic acid onto lignin under an ultrasonic field yielded PheLigNPs. The formation of PheLigNPs was evaluated by measuring the increase in phenols and the size of the resulting particles. Unmodified bulk lignin in suspension formed large microparticles observable by DLS with a phenolic content of 212.5 ± 5 mg GAE·g^–1^, while after the sonoenzymatic treatment, the phenolic content increased to 296.4 ± 14 mg GAE·g^–1^ and the size of the particles was on the nanoscale (Table S1, Supporting Information). This increase in the number of phenols confirmed the grafting of tannic acid onto lignin, while the size of the particles confirmed the nanotransformation. TEM images revealed that the PheLigNPs with an average size of 217 ± 54 nm were formed by smaller clusters (Figure 1). The cavitation phenomena caused by the application of ultrasound waves can produce changes in lignin macromolecules, disintegrating mechanically the aggregated particles to the nanoscale level.^11,12^ During this process, lignin most probably rearranged into NPs due to π–π interactions, van der Waals forces, and chain entanglement.^26−28^ The NPs maintained their size, PDI, and ζ-potential after 6 months of storage at 4 °C, indicating their high stability (Table S2, Supporting Information).

**Figure 2:**
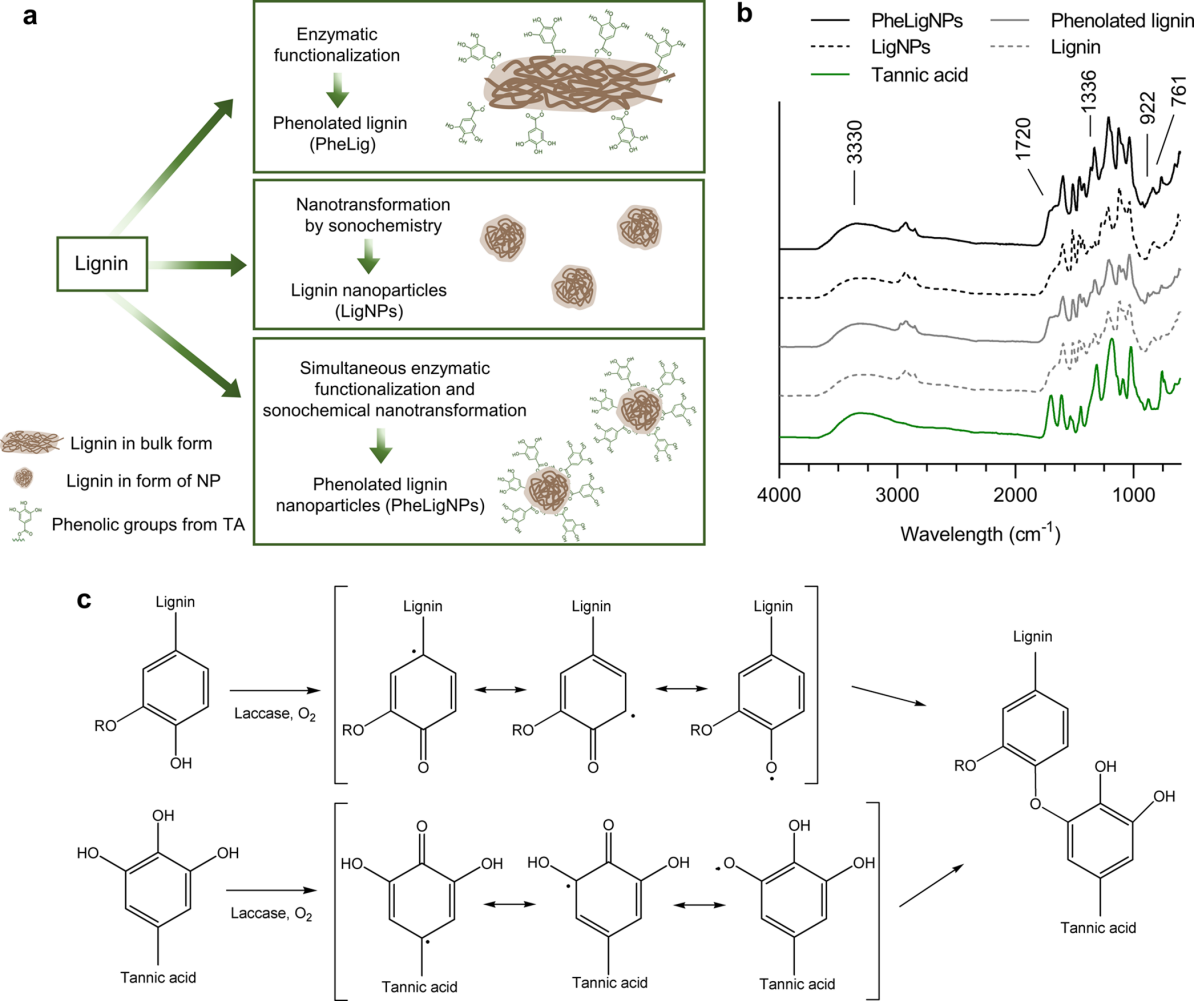
(a) Schematic representation of (1) phenolated lignin, (2) lignin nanoparticles, and (3) phenolated lignin nanoparticles. (b) FTIR spectra of all of the lignin samples and tannic acid. (c) Cross-linking reaction of lignin and tannic acid during laccase-assisted phenolation.

To study the contribution of the nanoform and the phenolic content to the antibacterial activity of the PheLigNPs, nonfunctionalized lignin nanoparticles (LigNPs) and phenolated bulk lignin (PheLig) were prepared and characterized (Figure 2a). PheLig presented a large particle size (>4000 nm) and phenolic content of 296.4 ± 14 mg GAE·g^–1^, while LigNPs presented a hydrodynamic size of 292 nm and a phenolic content of 222.92 ± 18 mg GAE·g^–1^ (Table S1 and Figure S1, Supporting Information).

Enzymatic phenolation of lignin was further characterized by FTIR to confirm that tannic acid was grafted onto lignin (Figure 2b). The characteristic bands of lignin appeared also in PheLigNPs, LigNPs, and phenolated lignin, reflecting presence in the samples of the primary subunits of lignin, namely guaiacyl (G), syringyl (S), and p-hydroxyphenyl (H).^29^ Upon phenolation, an increase in the intensity of the O–H stretching band at 3000–3600 cm^–1^ in the phenolated samples (PheLigNPs and PheLig) was observed, indicating an increase in the phenolic hydroxyl groups following the reaction with tannic acid.^15,16^ This band was also observed in the tannic acid spectra due to the presence of hydroxyl groups. The intensity of the O–H stretching band was slightly higher in the LigNPs than that in the unmodified lignin, which was attributed to partial oxidation of lignin induced by the ultrasonication process.^12,30^ The signal at 1720 cm^–1^, corresponding to the C=O stretching vibration of unconjugated carbonyl groups,^31^ was observed as a sharp peak in tannic acid and as a shoulder in the lignin samples. This peak was more accentuated in PheLigNPs and PheLig compared to that in their nonphenolated counterparts, confirming the presence of grafted tannic acid. Further evidence for the successful phenolation of lignin is the appearance of the absorption band at 1366 cm^–1^ in PheLigNPs spectra corresponding to the presence of phenolic O–H.^24,32,33^ New characteristic peaks appeared at 922 and 760 cm^–1^, associated with the C–H out-plane flexural vibration on aromatic rings.^15,16,32^ A strong band at 756 cm^–1^ was also observed in the tannic acid spectrum. The changes in these absorption bands were corroborated by the *A*_x_/*A*_2920_ ratio (Table S2, Supporting Information). These observations indicate that lignin’s phenolic groups are oxidized to phenoxy radicals by the action of laccase^22^ and then undergo coupling reactions cross-linking with tannic acid molecules, thus increasing the amount of phenolic groups (Figure 2c).

### Antibacterial Activity of PheLigNPs

In this work, plant-derived polyphenol tannic acid was used as a functional molecule to increase the antibacterial activity of lignin NPs. The antibacterial activity of the PheLigNPs was determined by a standard broth dilution method against Gram-positive *S. aureus* and *B. cereus* and Gram-negative *P. aeruginosa* and *E. coli*, which are relevant pathogenic bacteria causing medical- or food-related infections. To elucidate the factors contributing to the antibacterial effect of PheLigNPs, i.e., phenolic content and nanoform, the antibacterial activity of nonfunctionalized lignin NPs (LigNPs), functionalized lignin in the bulk form (PheLig), and pristine lignin was also studied. The MIC of PheLigNPs was 1.25 mg·mL^–1^ for *S. aureus* and *B. cereus* and 2.5 mg·mL^–1^ for *P. aeruginosa* and *E. coli*, while the MICs of LigNPs, PheLig, and lignin for these bacteria were at least 2 times higher (Table 1). These results indicated that PheLigNPs presented higher antibacterial activity than their nonfunctionalized or bulk counterparts, which was attributed to both the higher phenolic content and the nanoform of PheLigNPs.

**Table 1:** MIC Values (mg·mL^–1^) of PheLigNPs, LigNPs, PheLig, and Lignin Assessed Using the Broth Dilution Method

	*S. aureus*	*B. cereus*	*P. aeruginosa*	*E. coli*
PheLigNPs	1.25	1.25	2.50	2.50
LigNPs	5.00	2.50	5.00	5.00
PheLig	>5.00	5.00	>5.00	5.00
Lignin	>5.00	5.00	>5.00	>5.00

**Figure 3:**
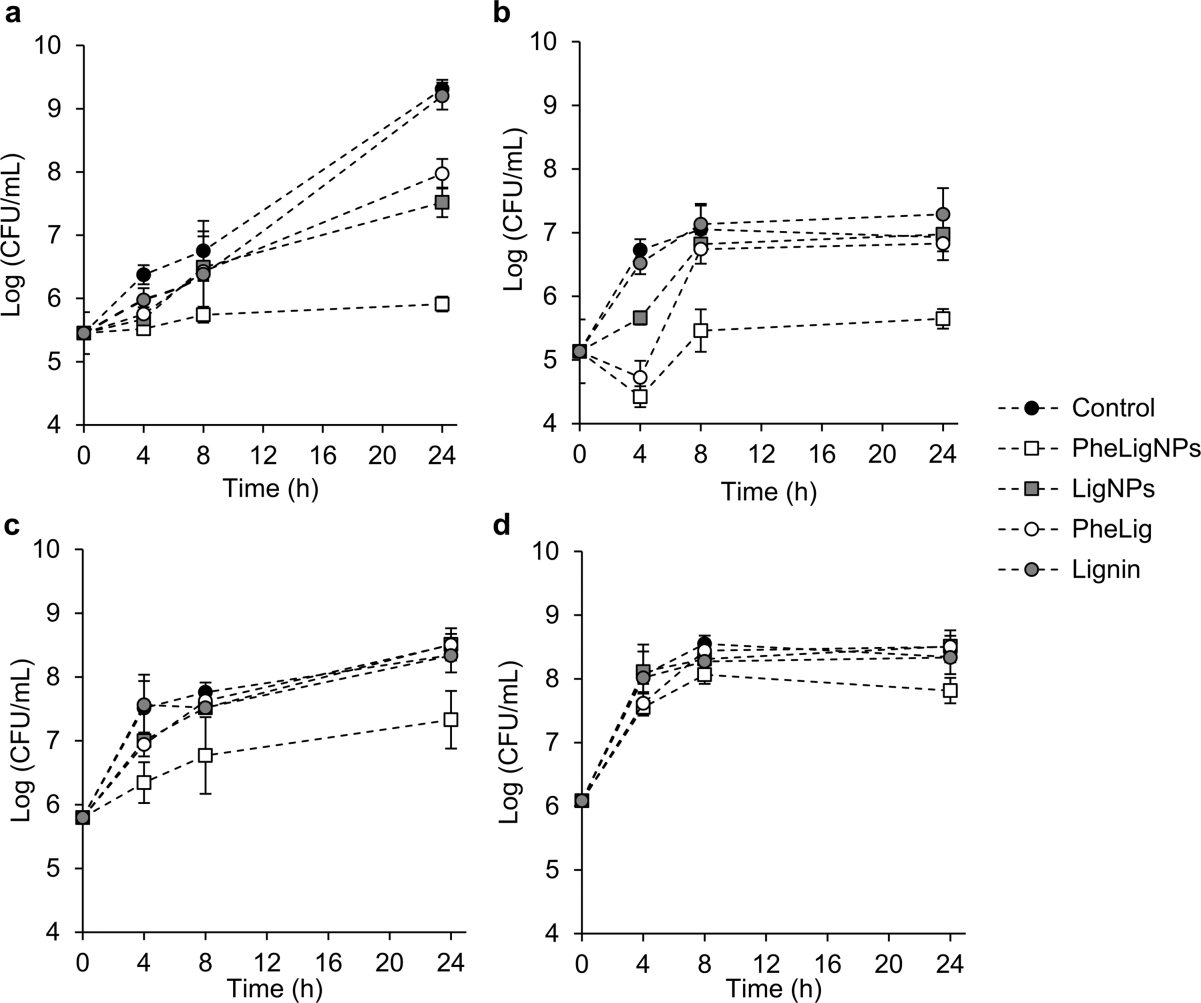
Kinetic growth curves of bacteria incubated with lignin samples at concentrations corresponding to MIC: (a) *S. aureus* (1.25 mg·mL^–1^), (b) *B. cereus* (1.25 mg·mL^–1^), (c) *P. aeruginosa* (2.5 mg·mL^–1^), and (d) *E. coli* (2.5 mg·mL^–1^). Results are reported as mean values ± standard deviation (SD) (*n* = 3).

Further evidence of the improved antibacterial effect of PheLigNPs was obtained with the kinetic growth curves of bacteria. PheLigNPs at MIC (1.25 mg·mL^–1^) were capable of inhibiting the growth of *S. aureus* and *B. cereus* (Figure 3a,b), as previously observed in the broth dilution method, while LigNPs, PheLig, and lignin at the same concentration as PheLigNPs did not induce such growth inhibition. The antibacterial effect of PheLigNPs against Gram-negative *P. aeruginosa* and *E. coli* was less pronounced, even at a higher concentration of NPs (2.5 mg·mL^–1^) (Figure 3c,d). However, the growth rate of *P. aeruginosa* was reduced when it was incubated with PheLigNPs compared to the growth of this bacteria in contact with other lignin samples. The observation of the poor activity of lignin and plant polyphenols against Gram-negative bacteria has been previously reported.^34,35^ The different susceptibility of Gram-positive and Gram-negative bacteria to these compounds is still unclear since other studies did not find a correlation between the antibacterial effect of polyphenols and Gram staining.^36−38^

### Cytotoxicity Assays with Human Cell Lines

**Figure 4:**
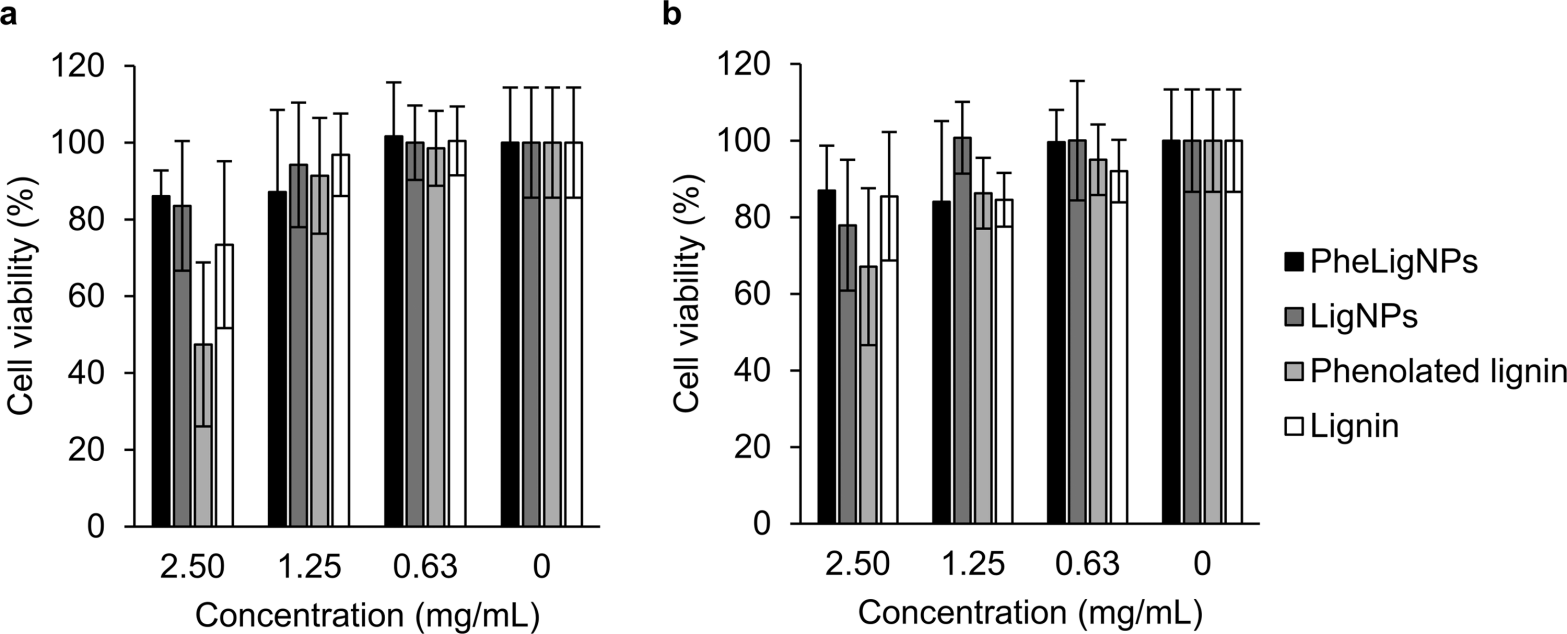
Cell viability (%) of (a) human keratinocytes and (b) fibroblasts exposed to PheLigNPs, LigNPs, PheLig, and lignin for 24 h assessed by the AlamarBlue assay. Results are reported as mean values ± SD (*n* = 3).

A crucial requirement for the biomedical translation of nanomaterials is their biocompatibility. Natural plant-based molecules have been used to develop bioactive NPs with low toxicity, demonstrating suitability as active agents for biomedical applications.^39^ The cytotoxic effects of PheLigNPs were assessed *in vitro* using human cell lines (Figure 4). After 24 h incubation with PheLigNPs at antibacterial concentrations (2.50–1.25 mg·mL^–1^), the cell viability of keratinocytes and fibroblasts was above 80% after incubation. Hence, the PheLigNPs are not considered toxic for human cells^40^ and have potential as antibacterial agents for applications in the field of biomedicine.

### Antibacterial Mechanism of Action of PheLigNPs

**Figure 5:**
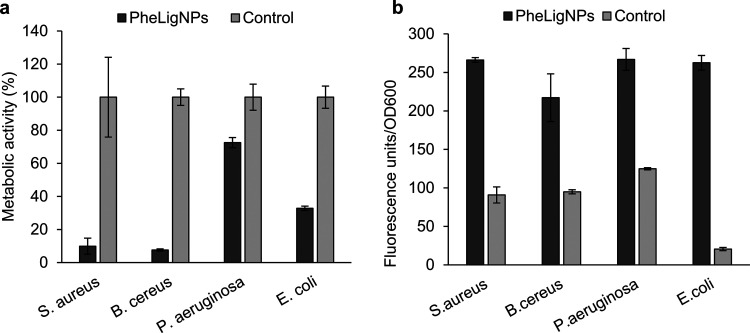
Antibacterial mechanism of action of PheLigNPs. (a) Evaluation of relative metabolic activity (%) of bacteria incubated with PheLigNPs by the resazurin assay. (b) ROS generation by bacteria in contact with PheLigNPs, assessed with fluorescent probe H_2_DCFDA. Controls refer to bacteria grown without PheLigNPs. Results are reported as mean values ± SD (*n* = 3).

The antibacterial mechanism of phenolic compounds is mainly attributed to (i) their ability to generate hydrogen peroxide, which coupled with metal ion complexation capacity, results in the inhibition of the activity of essential enzymes,^41^ and (ii) their ability to destabilize bacterial membrane, causing an increase of its permeability.^37,42^ In this work, the antibacterial mechanism of action of PheLigNPs was investigated by measuring the metabolic activity of bacteria and the generated ROS under the presence of PheLigNPs, and studying the interaction of the NPs with the bacterial membrane. Resazurin was used to determine the metabolic activity of bacteria incubated with subinhibitory concentrations of the NPs. Resazurin is a blue dye, which itself is nonfluorescent until it is reduced to the pink and fluorescent resorufin. In the cell, resazurin can be reduced by the activity of the bacterial respiratory chain. Bacteria incubated with PheLigNPs presented a decrease in metabolic activity compared to the control (Figure 5a). Since subinhibitory concentrations of PheLigNPs were used for this assay, the lower fluorescence levels detected were attributed to the low metabolic activity of viable bacteria. Hence, bacterial metabolism was reduced after the incubation of PheLigNPs.

Despite phenolic compounds being widely recognized as antioxidants, under certain conditions phenols can exhibit pro-oxidant behavior.^43^ Indeed, polyphenols have shown the ability to react with dissolved oxygen, resulting in the generation of hydrogen peroxide, which is involved in the antibacterial activity of the phenolic molecule.^41^ In this work, the ability of PheLigNPs to induce ROS in bacteria was studied using the fluorescent probe H_2_DCFDA (Figure 5b), which is activated by intracellular oxidants including hydrogen peroxide and the hydroxyl radical.^44,45^ Incubation of bacteria with the NPs resulted in an increase of ROS causing oxidative stress in the cell, which would result in lipid peroxidation, DNA damage, and enzyme inactivation.^46^

**Figure 6:**
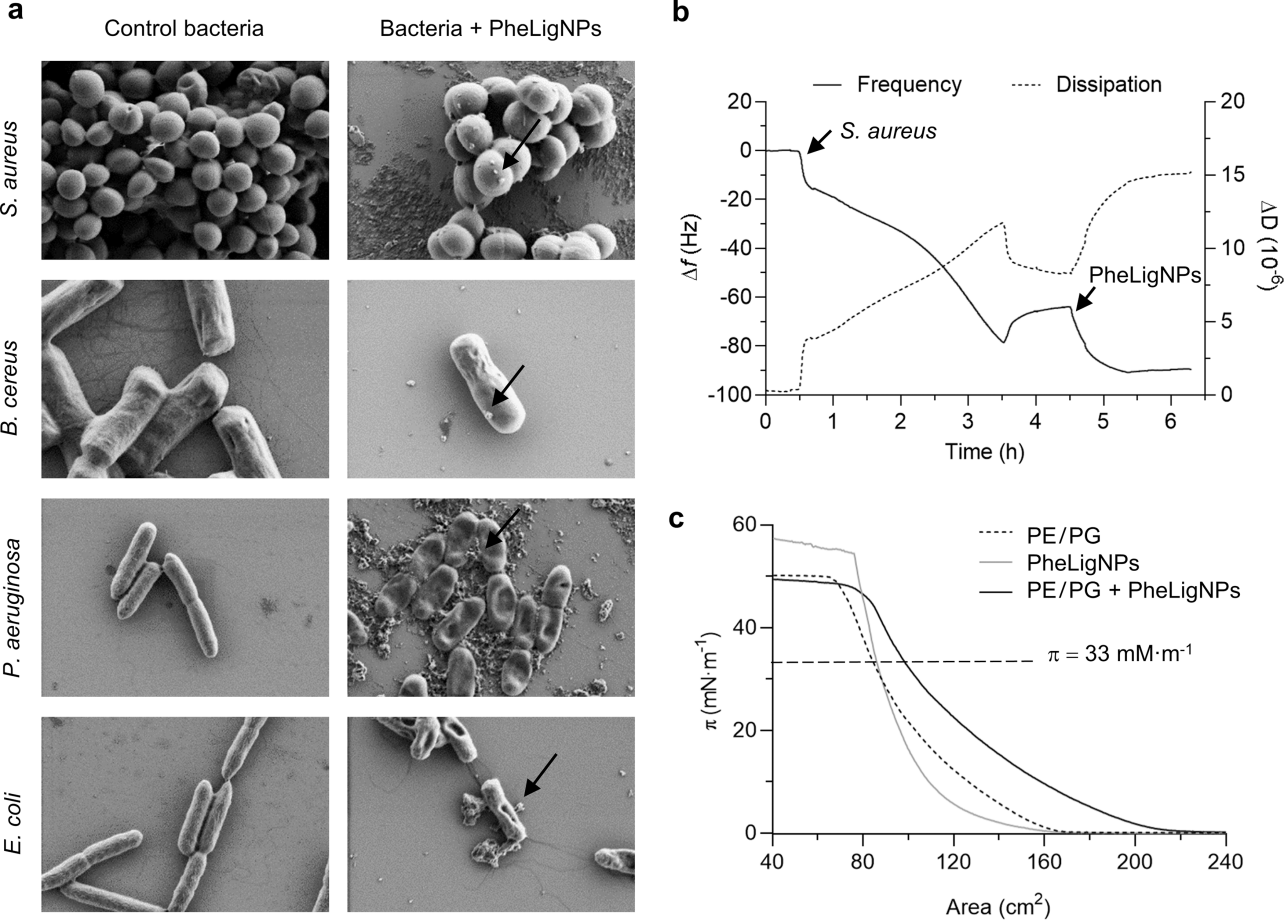
Study of the interaction of the PheLigNPs with bacteria. (a) SEM images of *S. aureus*, *B. cereus*, *P. aeruginosa*, and *E. coli* before and after exposure to PheLigNPs. (b) QCM-D monitoring of normalized frequency (Δ*f*) and dissipation (Δ*D*) obtained during the formation of a layer of *S. aureus* and its interaction with PheLigNPs. (c) Surface pressure–area isotherm of a PE/PG mixture monolayer in PBS, PheLigNPs in PBS, and PE/PG in PBS with PheLigNPs.

Ultrastructural analysis of bacteria incubated with PheLigNPs allowed the study of the NP–bacteria interaction and the morphological changes in bacteria after being exposed to the NPs (Figure 6a). Control bacteria, which were grown in normal conditions, presented undamaged membranes. When bacteria were incubated with PheLigNPs, the NPs were attached to the bacteria, suggesting a possible interaction with the bacterial surface. *S. aureus* and *B. cereus*, the two Gram-positive bacteria, presented changes in the surface roughness, but their shape did not present significant alterations in comparison to the control. Gram-negative *P. aeruginosa* and *E. coli*, however, did change their morphology upon incubation with PheLigNPs. Despite cellular lysis or membrane cleavage not being observed, these bacteria were flattened, presenting several depressed areas.

Further evidence of the interaction between PheLigNPs and bacteria was obtained by QCM-D (Figure 6b), which provides real-time outputs of molecular adsorption and interactions taking place on the sensors. After establishing an initial baseline with PBS, *S. aureus* was circulated until its deposition on the sensor, which resulted in a constant frequency decrease coupled with an increase in dissipation. After removing the unbound materials by circulating PBS, a steady-state frequency attributed to the deposited bacteria was measured. Hence, the presence of *S. aureus* in the sensor was confirmed by the difference in the frequency between the initial and the second PBS baselines (≈65 Hz). The circulation of PheLigNPs followed by PBS caused a progressive frequency decrease (≈25 Hz) and dissipation increase, which was attributed to the interaction of PheLigNPs with bacteria and their subsequent deposition. The adhesion of PheLigNPs onto bacterial surfaces might be caused by multiple interactions between the phenolic moieties of the particle and the components of the cell envelope. According to previous studies, phenolic groups can interact with amino and thiol groups from membrane proteins,^47^ and due to their hydrophobicity, phenolic moieties can interact with lipid membranes.^48^

The interaction of PheLigNPs with a mimetic Gram-negative bacterial model membrane was investigated using Langmuir isotherms. The two main phospholipids of the *E. coli* outer membrane, PE and PG, were used to form a monolayer. The PE/PG surface pressure–area isotherm (Figure 6c) presented a monotonic increase until the collapse pressure at ∼49 mN·m^–1^, as previously reported.^49^ The PheLigNPs isotherm evidenced their high surface activity, which might contribute to their capacity to interact with bacterial membranes. Indeed, the isotherm of the model membrane containing PheLigNPs (PE/PG + PheLigNPs) was displaced to larger areas in comparison with the PE/PG isotherm, and the collapse pressure decreased to ∼43 mN·m^–1^. Focusing on the physiological membrane pressure, 33 mN·m^–1^, PE/PG monolayers presented an area of ∼85 cm^2^, while in the PE/PG + PheLigNP isotherm, the area was ∼99 cm^2^. The increase in the area recorded can be attributed to the intercalation of the NPs between the phospholipid chains, probably due to their hydrophobic nature.^50^ Similar behavior has been reported for surface active lignin-capped silver NPs, which caused a membrane-disturbing effect.^14^

The integrity of the bacterial membranes was also studied using two fluorescent dyes: SYTO-9, which stains the cells with intact membranes in fluorescence green, and propidium iodide, which stains the cells in fluorescent red only after penetrating through damaged membranes. The control bacteria appeared mostly in green, revealing the integrity of their cytoplasmic membrane. Contrarily, when bacteria were incubated with PheLigNPs, red cells predominated, indicating that their membrane was damaged (Figure S2, Supporting Information). Moreover, the SYTO-9/propidium iodide fluorescence ratio was higher for control bacteria than that for bacteria incubated with PheLigNPs, indicating the increase in the number of damaged cells after the treatment (Table S3, Supporting Information). The obtained images and the SYTO-9/propidium iodide fluorescence ratio supported the membrane-disturbing effect of PheLigNPs observed by Langmuir studies.

On the basis of the reported evidence, we assume that PheLigNPs have an affinity to the bacteria surface, being capable of interacting with proteins and intercalating into lipids, leading to membrane disturbance, inhibition of the respiratory chain, and formation of ROS that result in oxidative damage in the cells and hamper bacterial proliferation. However, additional studies need to be performed to completely elucidate the antibacterial modes of action of phenolic NPs. The fact that the NPs affect the bacteria at different levels might hamper the acquisition of resistance by bacteria, which evidence the suitability of PheLigNPs as alternative plant-based antibacterial agents.

### Resistance Development

Since PheLigNPs have shown multiple antibacterial modes of action, we hypothesized that they might avoid the development of resistance in bacteria. AMR is a natural phenomenon taking place when bacteria acquire the capacity of tolerating a certain antimicrobial agent after long exposure. In the case of conventional antibiotics, their targets are specific pathways or molecules essential for the viability of bacteria. Under the selective pressure of an antibiotic, susceptible bacteria are inhibited or eradicated, while the cells that acquire antibiotic-resistant traits are able to survive. According to the World Health Organization, AMR is one of the main global health threats and needs to be urgently addressed.^51^ After exposing *S. aureus* and *E. coli* to conventional antibiotics for 30 days, the MIC significantly increased by 1281 for ciprofloxacin and by 96 for ampicillin (Table 2). This indicates that bacteria developed antimicrobial resistance in response to antibiotics. On the contrary, almost no increase in the MIC was observed when *S. aureus* was incubated with PheLigNPs (an increase of 1.5) and no change in the MIC was observed for *E. coli*, indicating that resistance was not developed. Efforts have been made to develop alternative bactericidal compounds such as AgNPs and metal oxide NPs; however, some reports already reported the surge of bacterial strains resistant to ionic silver and even AgNPs.^52^ Therefore, developing effective antibacterial agents that avoid the appearance of resistant strains is still challenging. Given the multiple and nonspecific antibacterial modes of action of the PheLigNPs, including direct contact with the bacterial surface, these NPs are less prone to induce resistance than traditional antibiotics.

**Table 2:** MIC Value Changes for *S. aureus* and *E. coli* Following 30 Day Exposure to Antibiotics or PheLigNPs

bacteria	antibiotic	PheLigNPs
*S. aureus*	1281 (ciprofloxacin)	1.5
*E. coli*	96 (ampicillin)	0

## Conclusions

In this work, laccase-assisted functionalization of lignin with plant-based phenolic compounds was combined with sonochemistry to produce PheLigNPs with enhanced antibacterial properties. This sonoenzymatic process yielded low polydisperse particles of 217 nm with an increased amount of phenolic groups in comparison with pristine lignin. The antibacterial effect of the particles was demonstrated against Gram-positive and Gram-negative bacteria typical for medical- and food-related infections. Both the higher phenolic content and the nanosize of the particles were responsible for the enhanced antibacterial effect of PheLigNPs in comparison with their nonfunctionalized or bulk counterparts. Studies on the antibacterial mode of action of PheLigNPs demonstrated the bacterial surface–particle interaction and membrane destabilization, coupled with increased levels of ROS and reduced metabolic activity. Bacteria in contact with PheLigNPs did not induce resistance, probably due to the multiple and unspecific targets, hence contributing to overcoming the growing concern of multidrug-resistant bacteria. The antibacterial activity together with their low cytotoxicity make these nanoparticles suitable for food packaging and biomedical applications.

## Supporting Information Available

Phenolic content, size, PDI, and ζ-potential of all of the lignin samples, TEM images and size distribution of LigNPs, peak analysis of the FTIR spectra, and fluorescence images and values of bacteria incubated with PheLigNPs (PDF)
