# Supplementary material for: Antibacterial Properties and Mechanisms of Action of Sonoenzymatically Synthesized Lignin-Based Nanoparticles
Source: ACS Appl Mater Interfaces. 2022 Aug 12;14(33):37270–9. doi: 10.1021/acsami.2c05443 (PMC9412960; doi:10.1021/acsami.2c05443)
Supplement: Supplementary file 1 — am2c05443_si_001.pdf [file am2c05443_si_001.pdf]

Supporting Information

Antibacterial properties and mechanism of action of sono-enzymatically synthesized lignin-based nanoparticles

Angela Gala Morena[†], Arnau Bassegoda[†], Michal Natan[‡], Gila Jacobi[‡], Ehud Banin[‡], Tzanko Tzanov[†]*

[†]Group of Molecular and Industrial Biotechnology, Department of Chemical Engineering, Universitat Politècnica de Catalunya, Rambla Sant Nebridi 22, Terrassa, 08222, Spain

[‡]The Mina and Everard Goodman Faculty of Life Sciences, Bar-Ilan University, Bldg 206, Ramat-Gan, 82900, Israel

*Corrsponding autor: Tzanko Tzanov, tel.: +34 93 739 85 70, fax: +34 93 739 82 25, e-mail: tzanko.tzanov@upc.edu

Table S1 Characterization of phenolated lignin nanoparticles (PheLigNPs), lignin nanoparticles (LigNPs), phenolated lignin (PheLig) and lignin: phenolic content measured according the Folin-Ciocalteu method, hydrodynamic size, polydispersity index (PDI), and ζ-potential.

	Phenolic content (mg GAE[a]/g sample)	Hydrodynamic size (nm)	PDI	ζ-potential (mV)
PheLigNPs	340.4 ± 16	293.5	0.293	-34.0 ± 0.5
LigNPs	222.92 ± 18	292.2	0.245	-34.4 ± 0.4
PheLig	296.4 ± 14	> 4000	1	-31.1 ± 0.7
Lignin	212.5 ± 6	> 4000	1	-32.2 ± 1.8

[a]GAE: gallic acid equivalents

Table S2 Stability of PheLigNPs and LigNPs assessed by measuring the hydrodynamic size, PDI, and ζ-potential after 6 months of storage at 4 ºC.

	Hydrodynamic size (nm)	PDI	ζ-potential (mV)
PheLigNPs	300.1	0.271	-32.6 ± 0.6
LigNPs	282.5	0.255	-31.2 ± 0.5

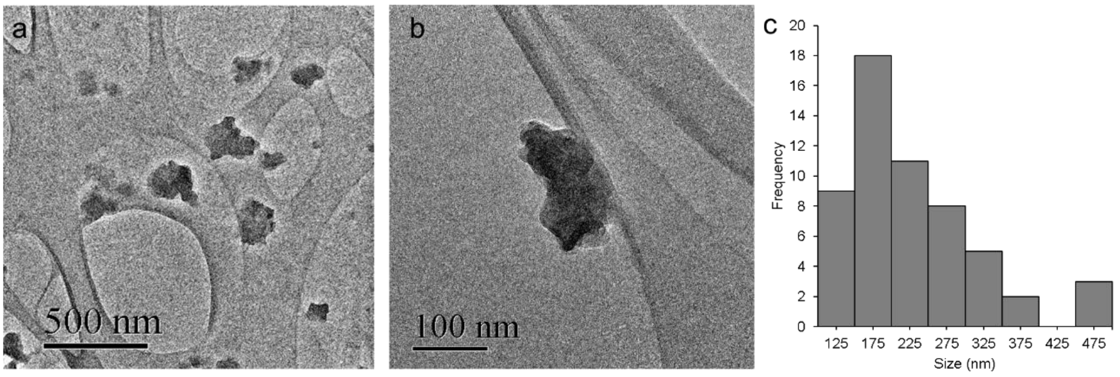

Figure S1. TEM images of LigNPs at (a) 6000X and (b) 30,000X magnification and (c) size distribution of the particles.

Table S2 Signal assignments and peak ratio of lignin samples in the FTIR spectra.

Assignments	Band position (cm^{-1})	A_x/A_{2920} ratio			
		Lignin	PheLigNPs	LigNPs	PheLig
Phenolic and aliphatic O-H stretching	3280	0.40	3.02	1.70	2.61
C-H stretching in aromatic methoxyl groups	2920	1.00	1.00	1.00	1.00
Phenolic hydroxyl groups	1366	0.58	1.62	0.44	0.56
Aromatic C-H out of plane bending	922	0.2	1.40	0.17	0.24
Aromatic C-H out of plane flexural vibration	761	0.84	1.90	0.02	0.85

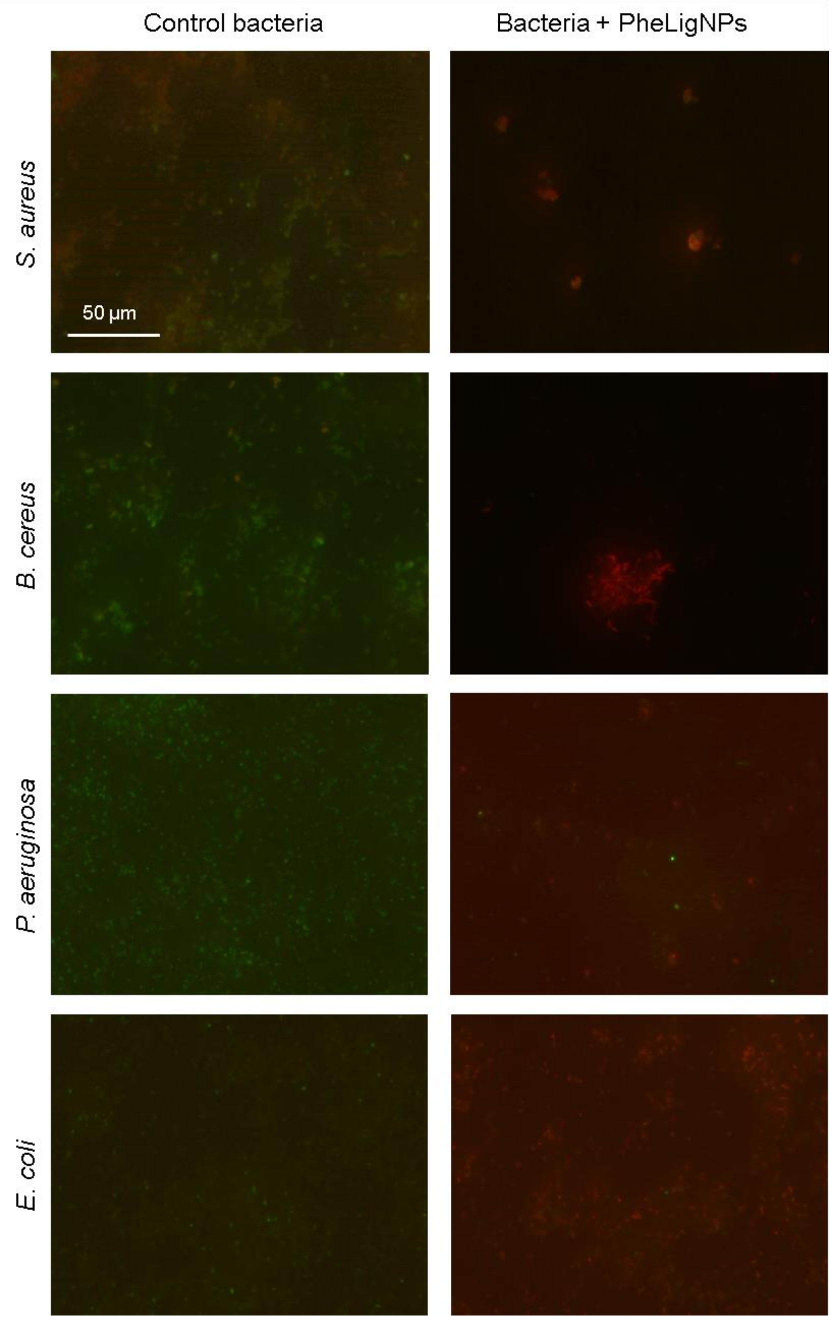

Figure S2 Fluorescence microscopy images of *S. aureus*, *B. cereus*, *P. aeruginosa*, and *E. coli* after 24 h treatment with PheLigNPs (0.6 mg/mL), or without NPs (control). Bacteria were stained using the green-fluorescent SYTO 9 that labels in green the bacteria with intact membrane, and red-fluorescent propidium iodide that labels bacteria with damaged membranes.

Table S3 Ratio of SYTO 9 to propidium iodide fluorescence emissions of bacteria incubated without PheLigNPs (control bacteria) and with PheLigNPs (bacteria + PheLigNPs) for 24 h.

	Control bacteria	Bacteria + PheLigNPs
S. aureus	34.8 ± 6.3	0.9 ± 0.3
B. cereus	7.8 ± 0.5	1.7 ± 0.2
P. aeruginosa	3.4 ± 0.4	1.1 ± 0.1
E. coli	8.4 ± 0.5	1.7 ± 0.2
